# Nutritional management of the child with chronic kidney disease and on dialysis

**DOI:** 10.1007/s00467-024-06444-z

**Published:** 2024-07-10

**Authors:** Vanessa Shaw, Caroline Anderson, An Desloovere, Larry A. Greenbaum, Lyndsay Harshman, Christina L. Nelms, Pearl Pugh, Nonnie Polderman, José Renken-Terhaerdt, Evelien Snauwaert, Stella Stabouli, Jetta Tuokkola, Johan Vande Walle, Bradley A. Warady, Fabio Paglialonga, Rukshana Shroff

**Affiliations:** 1 University College London Great Ormond Street Hospital Institute of Child Health, 30 Guilford Street, London, WC1N 1EH UK; 2 University Hospital Southampton NHS Foundation Trust, University of Southampton, Southampton, UK; 3 University Hospital Ghent, Ghent, Belgium; 4 Emory University and Children’s Healthcare of Atlanta, Atlanta, GA USA; 5 Stead Family Department of Pediatrics, University of Iowa, Iowa City, IA USA; 6 Children’s Mercy Kansas City, Kansas City, MO USA; 7 Queens Medical Centre, Nottingham Children’s Hospital, Nottingham, UK; 8 British Columbia Children’s Hospital, Vancouver, Canada; 9 Wilhemina Children’s Hospital, University Medical Center Utrecht, Utrecht, The Netherlands; 10 1st Department of Pediatrics, Aristotle University, Hippokratio Hospital, Thessaloniki, Greece; 11 Clinical Nutrition Unit, Internal Medicine and Rehabilitation, University of Helsinki and Helsinki University Hospital, Helsinki, Finland; 12 Fondazione IRCCS Ca’ Granda Ospedale Maggiore Policlinico, Milan, Italy; 13 University of Winchester, Winchester, UK; 14 Institute of Public Health and Clinical Nutrition, University of Eastern Finland, Kuopio, Finland

**Keywords:** Child, Nutrition, Growth, Energy, Protein, Phosphate, Chronic kidney disease

## Abstract

**Abstract:**

While it is widely accepted that the nutritional management of the infant with chronic kidney disease (CKD) is paramount to achieve normal growth and development, nutritional management is also of importance beyond 1 year of age, particularly in toddlers, to support the delayed infantile stage of growth that may extend to 2–3 years of age. Puberty is also a vulnerable period when nutritional needs are higher to support the expected growth spurt. Inadequate nutritional intake throughout childhood can result in failure to achieve full adult height potential, and there is an increased risk for abnormal neurodevelopment. Conversely, the rising prevalence of overweight and obesity among children with CKD underscores the necessity for effective nutritional strategies to mitigate the risk of metabolic syndrome that is not confined to the post-transplant population. Nutritional management is of primary importance in improving metabolic equilibrium and reducing CKD-related imbalances, particularly as the range of foods eaten by the child widens as they get older (including increased consumption of processed foods), and as CKD progresses. The aim of this review is to integrate the Pediatric Renal Nutrition Taskforce (PRNT) clinical practice recommendations (CPRs) for children (1–18 years) with CKD stages 2–5 and on dialysis (CKD2–5D). We provide a holistic approach to the overall nutritional management of the toddler, child, and young person. Collaboration between physicians and pediatric kidney dietitians is strongly advised to ensure comprehensive and tailored nutritional care for children with CKD, ultimately optimizing their growth and development.

**Graphical abstract:**

**Figure:**
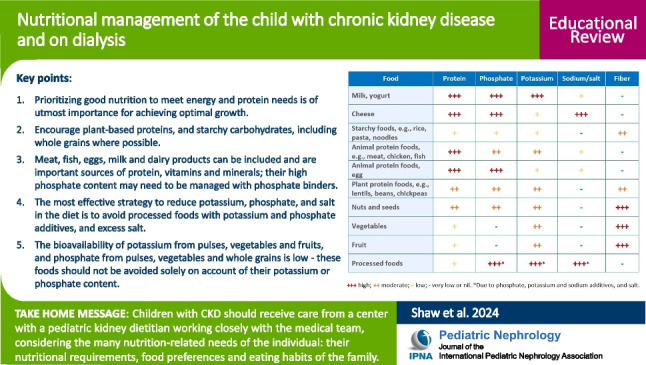
A higher resolution version of the Graphical abstract is available as Supplementary information

**Supplementary Information:**

The online version contains supplementary material available at 10.1007/s00467-024-06444-z.

## A holistic approach to nutrition for the toddler, child, and adolescent

For some children, their dietary prescription may necessitate adjustments for phosphate, potassium, salt, and possibly calcium content. In addition, the food that children eat must provide adequate energy, protein, vitamins, minerals, and fiber. We have published a number of CPRs for single dietary components but, given the multiple effects of CKD and the complex nutritional needs of children, it is impossible to act on a single nutrient without impacting other aspects of the diet. This educational review discusses how to integrate the CPRs in a practical way. For children with multiple nutritional needs, it is essential to engage the family by taking a holistic approach, considering the diet as a whole and not simply concentrating on nutrients as separate components of the diet. To successfully navigate these dietary complexities, families greatly benefit from the expertise of a pediatric kidney dietitian to guide them in providing a diet that is both manageable, enjoyable to eat, meets any necessary restrictions, and provides adequate nutrition. A multidisciplinary team should consistently communicate the same messages about nutrition to provide support to the family. Educational resources, whether verbal, written, pictorial, or digital, must be adapted to suit the learning style of the child and family so that they understand the need for any nutritional interventions. This understanding, in turn, will lead to better adherence and improved outcomes for the child with CKD.

## Nutrition, growth, and development

Inadequate nutrition is an important etiology of the poor growth seen in infants with CKD2–5D [1]. This, together with a high incidence of premature or small for gestational-age infants with CKD [2], often leads to suboptimal growth in children entering their second year of life. There is an opportunity for some catch-up growth in toddlerhood as the infantile phase of rapid growth may extend to 2–3 years of age [3]. Whereas optimizing the dietary prescription for the child with CKD is of vital importance at any age to promote growth and reverse growth failure if present, a further period of nutritional vulnerability occurs at puberty, even when growth is more dependent on the insulin-like growth factor-1 axis and sex steroids.

Achieving optimum nutritional management for the infant [4] has a beneficial outcome, not just on growth, but also on neurodevelopment, which continues into childhood. It is not known if poor nutrition and cognitive function in the pediatric CKD population are related. It is well-recognized that nutrition and inflammation can impact the development of learning and memory circuits. Notably, specific micronutrients such as iodine, zinc, vitamin B12, and iron have been correlated with neurodevelopment during the early to middle childhood phase [5]. A study of 34 infants who started long-term peritoneal dialysis (PD) aged ≤3 months concluded that aggressive nutrition, alongside other aspects of CKD management, contributed to a favorable developmental outcome [6]. The Chronic Kidney Disease in Children (CKiD) study showed that in children aged 6–16 years with mild to moderate CKD, a substantial percentage (21–40%) scored at least one standard deviation (SD) below the mean on measures of intelligence quotient, academic achievement, attention regulation or executive functioning, which puts them at risk for poor long-term educational and occupational outcomes [7]. A further study showed low academic achievement in over one-third of children with CKD, with the most difficulty observed in mathematics [8]. It is unknown how nutrition impacted these outcomes.

## Nutritional assessment

Before the dietary prescription of a child can be determined, it is necessary to conduct a nutritional assessment. The components of the nutritional assessment have been published by the PRNT [9] and include methods and tools for the assessment.

### Anthropometry

The key anthropometry to undertake is euvolemic (dry) weight, with adjustment of measured weight when indicated (e.g., when on dialysis or having nephrotic syndrome); recumbent length under 2 years of age, standing height thereafter, if able; and head circumference up to 2 years of age (or up to 3 years of age when appropriate centile charts are available). A child’s height is approximately 0.7 cm less than their length, so if recumbent length is used for a child over 2 years, this must always be recorded on their growth chart. The World Health Organization (WHO) growth charts [10] show this disjuncture between length and height on the 6-month to 2-year and 2 to 5-year charts. Arm span appears to be the most useful surrogate measurement for height in children who are unable to stand.

Growth parameters need to be measured routinely with the following minimum recommended frequency: for 1 to 3-year-olds with CKD3b–5 every 2 months and monthly for those with CKD5D; and every 3 months for older children and adolescents with CKD3b–5D. Where there are concerns about inadequate or excessive weight gain, measurements need to be undertaken more frequently. It is important to be aware that for children on dialysis, weight gain may result from fluid retention and be mistaken for the acquisition of body mass; indicators of excess weight due to fluid retention include edema on physical examination and hypertension that is responsive to fluid removal during dialysis. Other techniques, such as blood volume monitoring and bioelectric impedance analysis can be of help in assessing fluid excess, as clinical judgement may be misleading [9].

Measurements are only meaningful if plotted serially on centile growth charts. The PRNT recommends using WHO charts [10] or validated up-to-date country-specific growth charts, if available, as growth can vary with ethnicity. Disorder or genetic condition-specific growth charts can be used where growth potential may be altered, e.g., for those with trisomy 21 (Down syndrome), Wolf–Hirschhorn syndrome, and Prader–Willi syndrome [11–13]. While growth is at its maximum during the first year of life, the subsequent 11–12 cm gain in length during the second year must be supported by good nutrition. It is difficult to regain any loss in height centile after the age of 2 years, which has a consequent negative impact on final height potential. Kidney replacement therapy during childhood is associated with many impediments to catch-up growth, even with the use of growth hormone therapy [14, 15].

In the past, the nutritional focus on children with CKD has been related to their propensity for undernutrition and protein-energy wasting (PEW). However, the prevalence of overweight and obesity in this population has risen. In a study of 4474 patients from 25 European countries, aged less than 16 years, the prevalence of underweight was 3.5%, 20.8% were overweight, and 12.5% were obese [16]. The International Pediatric Peritoneal Dialysis Network (IPPN) registry documented a prevalence of 19.7% for overweight/obesity among children commencing chronic PD, compared with 8.9% underweight [17]. The CKiD study showed median energy intake to be higher than recommended in all age groups, implying that at least half the children consumed more energy than recommended [18]. The PRNT has published CPRs on the assessment and management of obesity and metabolic syndrome in children aged 2–18 years with CKD2–5D and post-kidney transplant [19]. It is recommended that anthropometry should be undertaken with a minimum frequency of every 1–3 months for children with CKD2–5 and transplant recipients and monthly for those with CKD5D. The following should be measured: euvolemic weight, length/height, weight-for-height (WFH), body mass index (BMI) for chronological age, and BMI-height-age, with standard deviation scores (SDS) calculated for each of these parameters. WFH should be plotted on WHO growth standard charts [10] for children aged 2–5 years: overweight is defined as WFH for age > +2 SD and obesity as WFH > +3 SD. For children over 5 years of age overweight is defined as BMI for age > +1 SD using WHO growth reference charts [10] or country-specific charts and obesity as BMI > +2 SD.

### Dietary assessment

Dietary assessment constitutes an integral part of the nutritional assessment of all children with CKD. Techniques for recording 3-day diet histories and 24-h recalls are described elsewhere [20]. The recommended frequency of dietary assessment depends on the rate of growth, CKD stage, blood biochemistry, and gastrointestinal symptoms. Evaluating appetite serves as a valuable tool to gauge nutritional risk during each clinical consultation. The CKiD study found that when categorizing appetite as “very good,” “good,” “fair,” “poor,” or “very poor,” any characterization apart from “very good” increased the risk of hospitalization and visits to the emergency department. Furthermore, a diminished appetite correlated with lower ratings of quality of life [21].

### Biochemical assessment

The only validated biochemical tool to assist in nutritional assessment is the normalized protein catabolic rate (nPCR) in adolescents receiving chronic hemodialysis (HD). nPCR values above 1 g/kg per day were associated with better growth, but there is no evidence for younger children, in whom a very high protein turnover may make this index less suitable. Serum albumin should only be utilized as a measure of nutritional status after excluding non-nutritional causes of hypoalbuminemia such as inflammation, infection, and fluid overload [9].

## The dietary prescription

### Energy and protein requirements

Achieving energy and protein requirements is fundamental to providing adequate nutrition and is the cornerstone of the dietary prescription. The evidence and rationale for energy and protein requirements have been described by the PRNT [22], using a novel term for dietary requirements, the suggested dietary intake (SDI). The lower and upper limits of the SDI for energy fall within the average daily amount of energy (i.e., the amount of energy considered sufficient to meet the needs of half the population) given in various international publications. The lower and upper limits of the SDI for protein fall within published protein requirements, which are average daily amounts +2 SD (i.e., the daily amount of protein considered sufficient to meet the needs of nearly all (97.5%) of the population) (Table 1). For children who exhibit suboptimal growth, reference to the SDI for height age (the age that aligns with a child’s stature when plotted on the 50th centile on a growth chart) may be appropriate. The SDI may be used for formulating the dietary prescription and assessing the adequacy of dietary intake for the individual child.

**Table 1:** Suggested Dietary Intake (SDI) for energy and protein

Age (years)	SDI* energy (kcal/kg/day)		SDI protein** (g/kg/day)	SDI protein (g/day)
	Male	Female		
1	72–120	72–120	0.9–1.14	11–14
2	81–95	79–92	0.9–1.05	11–15
3	80–82	76–77	0.9–1.05	13–15
4–6	67–93	64–90	0.85–0.95	16–22
7–8	60–77	56–75	0.9–0.95	19–28
9–10	55–69	49–63	0.9–0.95	26–40
11–12	48–63	43–57	0.9–0.95	34–42
13–14	44–63	39–50	0.8–0.9	34–50
15–17	40–55	36–46	0.8–0.9	Male: 52–65; Female: 45–49

For children with poor growth, reference to the SDI for height age may be appropriate

^*^SDI is based on the Physical Activity Level (PAL) used by international guidelines: 1–3 year PAL 1.4, 4–9 year PAL 1.6, and 10–17 year PAL 1.8. Where guidelines have given a range of energy requirements for different levels of PAL, the lowest PAL has been taken for SDI energy in consideration that children with CKD are likely to have a low activity level

^**^additional protein is required for those on dialysis: 0.15–0.3 g/kg/day for peritoneal dialysis; 0.1 g/kg/day for hemodialysis [23]

Twenty-three observational and retrospective studies have reported the effects of specified energy intakes in children with CKD [22], with most finding dietary energy intakes of around 100% of estimated energy requirements in those managed conservatively or on dialysis resulted in acceptable growth. Four trials studied resting energy expenditure or basal metabolic rate by indirect calorimetry and showed no difference for those with CKD from healthy children, after adjustment for lean body mass [24–27]. Consequently, the PRNT recommends that the initial prescription for energy intake should approximate that of the healthy child. In those with suboptimal weight gain and linear growth, the energy intake should be adjusted towards the higher end of the SDI. For those who are overweight or obese, energy intake should be individualized to achieve weight loss or weight maintenance without compromising their nutrition [19].

Two studies have estimated energy contributions from dialysate in children on PD (7.5 ± 7 and 9.08 ± 4.13 kcal/kg/day, respectively) [28, 29], and it may be important to include this in the calculation for total energy intake, especially in those who are overweight. However, it is important to be aware of the wide variation between patients due to differences in dialysate glucose concentration, number of cycles, dwell times, and peritoneal membrane transporter status.

Dietary protein restriction in the early stages of CKD should be avoided as a low protein intake may increase the risk of malnutrition, poor growth, and PEW. Inadequate protein not only impacts linear growth, but also affects body composition, with a preponderance of fat laid down rather than lean tissue [30]. Notably, children with CKD are known to have discordant body composition with high fat mass, low lean body mass, and increased central adiposity [31]. To foster optimal growth, it is strongly recommended that dietary protein intake should be targeted at the upper end of the SDI. The ranges for protein SDI outlined in Table 1 are appropriate for the initial dietary prescription. The protein intake at the lowest end of the range is considered the minimum safe amount [30]. Deviating below this threshold for protein may impede growth.

To compensate for protein losses in the dialysate, ranging from 0.28 g/kg/day in infants to 0.1 g/kg/day in adolescents [32], it might be necessary to consider protein intake levels above the SDI for individuals undergoing PD. The individual’s dietary protein needs can also be influenced by the peritoneal membrane transport characteristics, and transiently, by the increase of peritoneal protein losses that occur during peritonitis. The recommendations for additional protein provided by the National Kidney Foundation Disease Outcomes Quality Initiative (KDOQI) in 2009 [23] are still valid: 0.15–0.3 g/kg/day for PD; 0.1 g/kg/day for HD.

Children who are eating well may have a protein intake above the upper end of the SDI range which is not a problem *per se* if it does not result in unacceptably high urea levels. However, high protein intake may negatively affect acid-base balance and is frequently accompanied by a high phosphate intake which may lead to hyperphosphatemia and hyperparathyroidism. A study of 20 children on PD showed that dietary protein intake was negatively correlated with plasma bicarbonate, total body bone mineral density, bone mineral content, and fat-free mass [33].

It is expected that children with CKD have elevated urea levels. However, higher levels than expected for the child’s glomerular filtration rate (GFR) may indicate excessive dietary protein relative to energy intake. Prior to initiating any changes to the diet prescription, careful consideration of variables influencing urea levels is key, such as acute or chronic dehydration or steroid therapy. High urea levels may also be the result of a catabolic state consequent to inadequate energy intake; in this case, an increase in non-protein dietary energy may reduce urea levels. If the latter intervention is not sufficient and no other reason for the elevated urea level is detected, then protein intake may be reduced, keeping in mind the minimum protein requirements for adequate nutrition and growth, while preserving energy intake. If these dietary interventions do not reduce urea levels sufficiently, children with CKD stage 5 may require dialysis to address uremia, especially if symptomatic. However, some children tolerate chronically high urea levels. Conversely, levels that are lower than expected for the stage of CKD may indicate insufficient dietary protein.

### Vitamins, minerals, and trace elements

To complete the nutritional adequacy of the dietary prescription, it is important to provide enough vitamins, minerals, and trace elements; little is known about the requirements for children with CKD. The PRNT has published clinical practice points for the assessment and management of vitamin status in children with CKD2–5D and post-transplantation [34]. Vitamin requirements in early CKD (stages 2–3a) should approximate those of healthy children of the same chronological age. For children with advanced CKD (stages 3b–5D&T), vitamin requirements may be lower or higher than those for healthy children. Routine biochemical assessment of vitamin status is not indicated, except for vitamin D.

Dietary intervention or supplementation is warranted if dietary assessment reveals deficiency or excess, or if clinical and/or biochemical evidence suggests a risk of deficiency or excess. For children who are vitamin deficient, who are experiencing vitamin losses in dialysate, or who are taking medications that may interfere with vitamin metabolism, a water-soluble vitamin supplement may be required, particularly vitamins C, B6, and folate. The benefits of vitamin C supplementation must be weighed against the risks of its metabolism to oxalate. Folate and vitamin B12 supplementation should be considered in children with macrocytic anemia and biochemical evidence of deficiency. The intake of vitamin A in CKD2–5D, where accumulation may occur and is associated with hypercalcemia and raised intracranial pressure, should not exceed the requirements for healthy children. Consumption of vitamin A-rich food sources should be limited, and if nutritional supplements or formulas are given, those with a lower vitamin A content should be considered. If a multivitamin supplement is indicated, opting for a formulation with minimal or no vitamin A is advisable. There is no need for routine supplementation with vitamins E and K unless there is a comorbidity predisposing to deficiency, such as malabsorption.

Vitamin D deficiency is common in CKD. Evaluation, prevention, and treatment of vitamin D deficiency are complex, and this vitamin was not addressed by the PRNT [34] as assessment of vitamin D status, the optimal concentration of 25-hydroxyvitamin D, and indications for supplementation of native and active vitamin D supplementation are described elsewhere [35, 36]. It is noteworthy that pediatric enteral formulas contain a significant amount of vitamin D (around 1.0 micrograms/100 mL) which must be taken into account, if used, prior to prescribing supplemental vitamin D.

For minerals and trace elements, the advice provided by KDOQI [23] is reasonable to follow: at least 100% of the dietary reference intake for healthy children, with supplementation if dietary intake alone is insufficient.

## Feeding the child with CKD

### Eating and drinking

Breastfeeding is the preferred method for feeding an infant with CKD and can continue beyond the age of 1 year if the mother and child wish [37]. A significant advantage of breastmilk for the young child with CKD is its lower renal solute load, notably its low phosphate and potassium contents. Similarly, whey-dominant infant formula, with its protein and electrolyte content close to that of breastmilk, may be beneficial beyond the first year of life, particularly as it provides the range of vitamins and minerals necessary for overall health. From 1 year of age, Young Child Formulas (commercially available fortified milk drinks for toddlers, with low sodium, phosphate, and potassium content) may be useful, if available, as they contain iron, vitamin D, and omega-3 polyunsaturated fatty acids, which may enhance the quality of the diet.

Children with CKD should be offered a healthy, balanced diet with a wide variety of food choices, mirroring the same approach advocated for the general population, irrespective of their age or developmental stage. Meals should be based on the four food groups—starchy carbohydrates, fruits and vegetables, protein foods, and dairy products. Attention must be given to any foods that may need to be limited to correct elevated blood levels of urea, phosphate, and potassium and to reduce salt intake. Fresh foods are preferred over processed foods as this reduces the potassium and phosphate load of the diet provided by food additives and also reduces salt intake. Although high biological value protein foods such as meat, fish, eggs, and milk products provide the right proportion of essential amino acids for growth and protein repletion and have been favored in Western diets for children with CKD, there is a current shift with emphasizing the incorporation of plant-based, low biological value protein foods as they provide an important source of fiber. The incomplete profile of essential amino acids in individual plant foods (legumes and pulses including peas, beans, lentils, dhals; and grains (cereals) such as rice, pasta, couscous) can be remedied by combining pulses with cereals at mealtimes. Other plant foods (nuts and seeds; potatoes and other starchy roots and tubers; fruits and vegetables) provide further fiber.

### Fiber

Dietary fiber, an integral component of a healthy diet, has shown benefits for various conditions in adults with CKD, including obesity, hypertension, type 2 diabetes, dyslipidemia, and metabolic acidosis. Studies on the benefits of a high-fiber, plant-based diet in children are few, but the promotion of such a diet is gaining traction [38]. Dietary fiber is just as important for children with CKD as for healthy children. Snauwaert et al. have recently reviewed the benefits of fiber [39], among which are the prevention of constipation and fewer gastrointestinal symptoms, reduced inflammatory state, and decreased production of gut-derived uremic toxins. Additionally, plant foods, with their relatively lower protein content, may be given preferentially over animal-based foods if there is a need for reduced protein intake in the case of rising blood urea levels. However, due to the lower bioavailability of non-animal protein, in order to achieve the right amount and balance of high-quality protein, 1.2–1.3 times higher protein consumption is needed for those following a vegetarian or vegan diet, respectively [22].

The PRNT recommends that fiber intake should approximate that of healthy children of the same chronological age and has uniquely proposed daily amounts of fiber to be consumed by children with CKD. The SDI for fiber is shown in Table 2. Whenever possible, the preference is for intrinsic fiber (non-starch polysaccharides, resistant oligosaccharides, and resistant starch naturally present in foods). However, circumstances might also warrant the inclusion of added fiber (fiber that is chemically or physically extracted, enzymatically modified, or synthetically derived) to achieve the daily requirements. A holistic approach is necessary when increasing fiber intake. There are concerns that a high-fiber diet may lead to a feeling of fullness, thus compromising energy and protein intakes; the latter must be prioritized above fiber requirements, particularly in those children with a poor appetite or those who require a higher energy intake. In those with persistent hyperkalemia, the potassium content of fruits and vegetables may need to be addressed, but these foods should not be routinely omitted as the bioavailability of potassium in high-fiber foods is decreased. Similarly, in those with hyperphosphatemia, the low bioavailability of phosphate in grains and pulses (peas, beans, lentils) allows these foods to be included in the diet due to their rich fiber content. A fiber intake lower than the SDI is acceptable in children with fluid restriction. The PRNT recommends a comprehensive evaluation of the child’s fluid and fiber intakes along with stooling habits, by using the Bristol Stool Chart, at every consultation.

**Table 2:** Suggested Dietary Intake (SDI) for fiber

Age (years)	SDI (g/day)	SDI (g/1000 kcal)
1–3	14–19	8.3–14
4–8	18–25	8.3–14
9–13	24–31 (male); 20–26 (female)	8.3–14
14–18	28–38 (male); 22–26 (female)	8.3–14.2

### Tackling poor appetite

Refined carbohydrates (sugar, jams, honey, syrups) and fats (oils, margarines, butter) are advised to be used sparingly in a “healthy” diet, but they have a place in the diet of children with CKD who often have a poor appetite and need the energy that these foods provide. These food items can be added to high nutritional value sweet and savory foods (Supplementary Table 1). Vegetable margarines and oils with a high omega-3 fat content are preferred, such as soya, walnut, or linseed oil, or high in monounsaturated fat, e.g., olive oil. Conversely, children who are obese, with or without metabolic syndrome, should avoid sweetened foods and drinks and foods high in saturated (animal) fat and be offered a diet composed primarily of fruits and vegetables, whole grains, low- or non-fat dairy products, pulses, fish and lean meat [19].

As with infants with CKD, children may be reluctant to eat for a number of reasons. It is important to explore with the child and their family the many factors impacting their willingness to eat. Consider that their diet may be unnecessarily restrictive, with multiple dietary prescriptions. Permitted foods may be few and poorly presented, with the additional issue of families having limited food choices to offer their child due to their cost and availability. Adolescents, in particular, find dietary modifications difficult to follow as they wish to eat with their peers and increasingly rely on “fast” processed foods. Dietary education may be delivered in an unattractive way which impacts a child’s willingness to comply with dietary advice.

There are medical causes for a poor appetite, including vomiting resulting from gastroesophageal reflux (GER), delayed gastric emptying, and gut dysmotility associated with decreased clearance of polypeptide hormones and cytokines [40, 41]. The presence of dialysis fluid can lead to a feeling of fullness and persistent vomiting, both of which can have a negative impact on appetite and contribute to nutritional inadequacy. An additional factor for those on PD is raised intra-abdominal pressure [42]. Acidosis, anemia, and uremia also negatively influence oral intake if not corrected. Moreover, children with CKD have reduced smell and taste sensations [43], which worsen as CKD progresses. Finally, the polyuric child preferentially drinks large amounts of water, and the many medications that may be required in CKD further reduce appetite. It is important to minimize the impact of these issues, where possible. GER may be treated with alginates, antacids, histamine H_2_ receptor antagonists, proton pump inhibitors, and prokinetics.

If the correction of the medical causes of poor appetite and intake have been addressed and weight and height gain are still suboptimal, as reflected by the child faltering on their growth centiles, nutritional intervention is needed. Foods and drinks can be supplemented with sugars and fats (Supplementary Table 1). The use of glucose polymers allows a greater amount of carbohydrate to be added to a given amount of water as they have a neutral taste and exert a lesser osmotic effect on the gut than sugar or glucose. Starting with 5% (5 g glucose polymer added to 100 mL) and increasing to 30% for older children and adolescents as tolerated and needed provides an extra 20–120 kcal per 100 mL of water. Combined fat/glucose polymer modules provide 5 kcal/g powder. Nutritionally complete oral liquids (oral nutritional supplements or sip feeds) are a good way to improve not only energy and protein intakes, but also provide vitamins and minerals to a diet that may well be deficient in these. If a child cannot achieve their requirements for energy and protein despite oral supplementation, the PRNT recommends supplemental or exclusive enteral tube feeding to improve their nutritional status.

### Enteral tube feeding

Intervention may be appropriate at any age. Eight retrospective studies (in which tube feeds were either supplemental to oral intake or provided up to 100% of energy and protein requirements) showed improvement in weight and BMI SDS in children <2 years and an improvement in height SDS in six of these studies. A study where tube feeding was commenced after 2 years of age in prepubertal children showed improvement in both height and weight SDS. Younger children and those not on dialysis had the greatest benefit [44]. There have been concerns that long-term enteral feeding (EF) results in obesity in some children, but a study of 40 children with CKD3–5D showed improvement in both weight and BMI SDS without leading to obesity [45].

The PRNT has published CPRs for EF in children with CKD2–5D [46]. A nasogastric (NG) tube is the preferred option for short-term EF, while a gastrostomy device (GD) is preferable for long-term EF. Preparations and investigations prior to insertion of a GD and techniques used for their insertion, especially for children who have a PD catheter *in situ*, are fully described, together with advice on feed delivery by gravity bolus or continuous feeding via gastrostomy and EF pump. Of note, continuous NG overnight feeding at home poses a significant risk for aspiration of the formula if used unsupervised; a local risk assessment should be conducted. For a child who is likely to need PD, and in whom enteral tube feeding is required, the GD should ideally be placed before insertion of the PD catheter to decrease the risk of peritonitis following GD placement [46, 47].

For young children requiring enteral tube feeding, concentrated whey-based infant formulas can be used (20 g infant formula powder plus cooled boiled water up to 100 mL) to provide an energy density of 1 kcal/mL, with due attention to adequacy of vitamins and minerals. Commercially available ready-to-feed energy and nutrient-dense infant formulas may be a suitable option; however, careful attention to the profile of each formula is warranted to ensure that nutrient needs are met and not exceeded. Standard and high-energy pediatric enteral formulas are designed to provide the sole source of nutrition for children aged 1–12 years, though the potassium, phosphate, and vitamin A content of these, and concentrated infant formulas, may be higher than desired for children with CKD. Standard adult enteral formulas are often used for adolescents (over 45 kg body weight); the vitamin and mineral contents need to be checked against requirements for this age group as they may be very high. Examples of typical enteral formulas are given in Supplementary Table 2. It is advisable to choose a formula with added fiber, usually present as fructo-oligosaccharides or soy polysaccharides. It is sometimes necessary to add extra energy to enteral formulas, in the form of carbohydrate and fat, for the catabolic child, or in children requiring a fluid restriction. Supplementary Table 3 gives guidance on how this can be done. Renal-specific formulas are described in the Potassium section. Caregivers may wish to prepare their child’s tube feeds by blending household foods. This must be done under the supervision of a qualified dietitian who can advise about safety issues including nutritional quality, microbial contamination, and appropriate equipment and administration [48].

Continuous EF provided overnight via GD can be helpful in reducing the persistent vomiting experienced by many children. This strategy involves administering small volumes of formula over an extended period thus facilitating the emptying of the dysmotile stomach. Anecdotal evidence suggests that children actually eat more food when on gastrostomy feeds than before starting EF, and this is likely attributed to reduced episodes of vomiting and an enhanced overall sense of well-being. Continuous feeding may lead to higher pH values in the stomach which can promote bacterial growth, particularly affecting those with significant dysmotility [49]. Sufficient time off the EF pump should be given to allow the stomach to return to its pre-prandial pH.

If suboptimal growth persists, despite the provision of adequate nutrition through supplementation of the oral diet or EF and despite good metabolic equilibrium, growth hormone therapy should be considered provided the child has growth potential [15].

## Modifying the dietary prescription

**Fig. 1:**
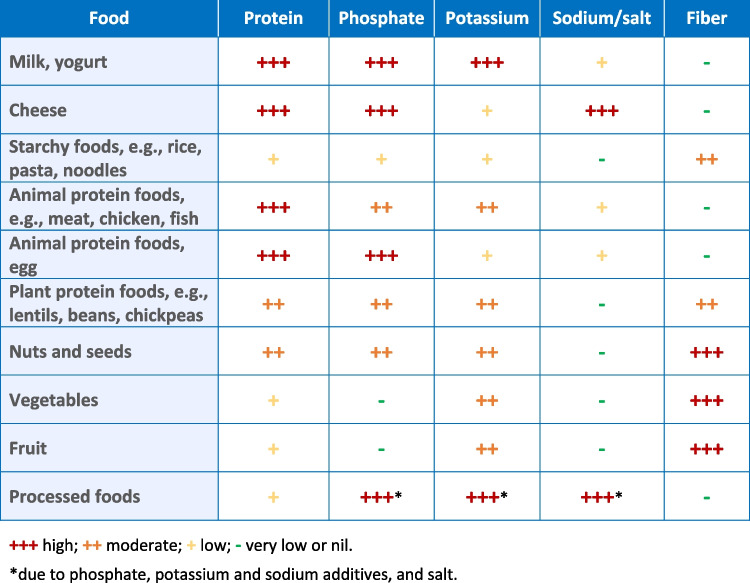
Guide to the nutrient content of foods. Caution: when advising the individual child consider first their blood biochemistry, diet as a whole, appetite and food preferences, portion sizes, food availability, and family’s cooking skills before restricting any nutrients

As CKD advances and the child matures and is exposed to a wider variety of foods, adjustments to the diet will be imperative. Food choices may need to be modified to maintain serum levels within age-appropriate normal ranges. Hyperparathyroidism secondary to phosphate retention may be seen as early as CKD stage 2 so it is advisable to start to restrict phosphate in the diet from an early age. Dietary salt restriction is beneficial for many in the early stages of CKD, reducing blood pressure and thereby slowing a decline in kidney function. Potassium excretion is not usually affected until the GFR falls below 15–20 mL/min/1.73 m^2^; however, even children with moderate CKD may develop hyperkalemia and require a reduction in potassium intake. A holistic approach is vital when modifying the diet to ensure that any restrictions are limited wherever possible so as not to worsen any pre-existing reluctance of the child to eat. While tackling intakes of phosphate, salt, and potassium it is important to ensure nutritional adequacy with sufficient intake of energy, protein, calcium, fiber, vitamins, and minerals. Table 3 shows food choices that can be made to accommodate the challenge of multiple dietary modifications. Figure 1 provides a quick guide to the nutrient content of foods. Any changes in the dietary prescription should be based on trends of serial biochemistry results rather than a single result and without compromising the child’s overall nutritional status.

**Table 3:** Modifying phosphate, potassium, and sodium/salt intakes. Portion size may need to be controlled to ensure foods containing phosphate and potassium meet the criterion of “lower option.” Cooking methods may reduce the phosphate and potassium content of foods

	Food type		
Lower phosphate options		Lower potassium options	Lower sodium/salt options—do not add salt in cooking or at the table
Breastmilk, whey-dominant infant formula, plant-based milks (e.g., almond, oat, soy)—avoid added calcium phosphate	Milk	Breastmilk, whey-dominant infant formula, plant-based milks (e.g., almond, oat, soy)	
Cottage, cream, ricotta cheese; paneer	Cheese, yogurt	Any type	Avoid processed cheese Lower salt: mozzarella, cream cheese
Rice, pasta, noodles, couscous, millet, semolina, tapioca, bulgur wheat, cassava, potatoes, plantain, quinoa, yam; bread, chapattis, pitta, tortillas; breakfast cereals	Starchy foods	Rice, pasta, noodles, couscous, millet, semolina, tapioca; bread, chapattis, pitta, tortillas; breakfast cereals Caution higher K: bulgar wheat, cassava, potatoes, plantain, quinoa, yam	Avoid salted potato crisps (chips); instant pasta, rice, and noodle meals; check the salt content of breakfast cereals
Fresh, unprocessed, any type	Meat, chicken, fish	Fresh, unprocessed, any type	Avoid processed foods
Egg white	Eggs	Whole egg	
Any type e.g., lentils, dhals, split peas, chickpeas; black eye, broad, butter, cannellini, red kidney beans*; tofu; houmous	Pulses	Lentils, dhals, split peas, chickpeas; cannellini, red kidney beans; tofu; houmous Caution higher K: black eye, broad, butter beans	Check canned foods do not have salt added
Any type e.g., almonds, hazelnuts, peanuts, walnuts; pumpkin, sesame, sunflower seeds; tahini paste	Nuts and seeds	Almonds, hazelnuts, peanuts, walnuts; sesame and sunflower seeds; tahini paste Caution higher K: pumpkin seeds	Avoid salted varieties
Any type e.g., avocado, broccoli, brussels sprouts, butternut squash, cabbage, carrot, cauliflower, courgette, okra, plantain, pumpkin, swede, sweet potato, tomato	Vegetables	Broccoli, butternut squash, cabbage, carrot, cauliflower, courgette, pumpkin, swede Caution higher K: avocado, brussels sprouts, okra, plantain, sweet potato, tomato	
Any type e.g., apple, apricot, banana, blueberries, lychees, mango, melon, orange, papaya, pear, pineapple, raspberries, strawberries	Fruits	Apple, blueberries, lychees, pear, pineapple, raspberries Caution higher K: apricot, banana, mango, melon, orange, papaya, strawberries	
Avoid phosphate additives – bioavailability up to 100%	Food additives	Avoid potassium additives—bioavailability 90–100%	Avoid salt and sodium additives

^*^Canned varieties have a lower phosphate content than dry pulses

The PRNT has published a full account of the nutritional management of calcium, phosphate [20], and potassium [50], and these are summarised below.

### Calcium

Calcium is necessary for bone mineralization and to prevent fractures [51]; therefore, a low dietary calcium intake is of concern. Conversely, a high calcium intake may lead to vascular calcification, progressive vessel stiffness, and left ventricular failure [52–56]. Calcium intake should provide at least 100% of the SDI (Table 4) and no more than twice the upper SDI value. Assessment of calcium from all sources (food, breast milk, formula, medications) is essential to ensure sufficient, but not excessive calcium intake. Insufficient dietary calcium, likely due to a restriction of dairy foods (e.g., milk, cheese, yogurt) imposed to reduce phosphate intake, was observed in 76% of 46 children with CKD4–5D [57]. This can be addressed by encouraging increased consumption of non-dairy calcium-rich foods such as green-leafy vegetables, beans, nuts and nut butters, and calcium-fortified foods such as bread and breakfast cereals. Calcium-based phosphate binders contribute to calcium intake and some children require calcium supplements for their intake to fall within the SDI range.

**Table 4:** Suggested Dietary Intake (SDI) for calcium and phosphate

Age (years)	SDI calcium (mg/day)	SDI phosphate (mg/day)
1–3 years	450–700	250–500
4–10 years	700–1000	440–800
11–17 years	900–1300	640–1250

In those with mineral-depleted bone, persistent hypocalcemia, or hyperparathyroidism, calcium intake may need to be increased above 200% SDI for a short period under close medical supervision [20]. Non-dairy calcium-rich foods can be given, but additional calcium through calcium supplementation and/or high calcium dialysate will often be necessary. Absorption of calcium requires adequate vitamin D, and supplementation is often necessary (usually both native and active forms) [35, 36]. Abnormalities in acid-base status, i.e., metabolic alkalosis due to frequent vomiting, or hypoalbuminemia, might affect the interpretation of serum calcium levels; ionized calcium levels must be measured so that hypocalcemia can be detected and managed appropriately. Importantly, serum calcium accounts for <0.1% of total body calcium and is not a good measure of total body calcium.

The management of persistent mild to moderate hypercalcemia is mainly medical: interventions include reducing or stopping calcium supplements and calcium-based phosphate binders, reduction or stoppage of native and active vitamin D, and use of a lower calcium dialysate. A temporary reduction of dietary calcium may also be necessary. Substituting milk in the diet with a specialized low-calcium infant formula or incorporating it into all, or part, of any enteral formula should be considered. Using deionized or distilled water also reduces calcium intake. Regular assessment of dietary calcium is required, especially when calcium intake is reduced below the SDI. Hypercalcemia may also be due to hypervitaminosis A and if detected, should prompt a reduction in vitamin A intake, but ensuring the maintenance of optimal nutrition.

### Phosphate

Hyperphosphatemia is very commonly seen in children with kidney failure and the IPPN registry showed that the prevalence of hyperphosphatemia increased with age from 6% in young infants to 81% in adolescents [58]. As hyperparathyroidism secondary to phosphate retention may be seen in CKD stage 2, dietary phosphate intake may need to be limited to within the SDI even in the mild to moderate stages of CKD. If there is either persistent hyperphosphatemia or persistent hyperparathyroidism, phosphate intake should be reduced to the lower end of the SDI range (Table 4) [20].

The single most effective dietary modification to reduce phosphate intake is the avoidance of processed foods containing phosphate additives. These are commonly added to meat and fish products (sausages, salamis, burgers, chicken nuggets, fish fingers), commercial bakery items (cakes, biscuits, crumpets, tortilla wraps, naan bread), potato products (frozen chips, waffles), dairy foods (milk desserts, yogurts, ice cream, cheese slice slices and spreads), dark-colored carbonated (fizzy) drinks, and chocolate- and malt-based drinks. It is crucial to educate children and their caregivers about the importance of reading food labels diligently and being vigilant about avoiding products that include “phos” in the ingredient list. The inorganic phosphate salts added to processed foods are not organically bound to, or associated with, phytate, resulting in an extremely high bioavailability of up to 100% [59–61]. Conversely, the phosphate in fresh (unprocessed) foods is carbon-bound, with the lowest bioavailability of 30–40% being in plant-based foods, where phosphate is stored in the form of phytate [62], which humans cannot digest. Although whole grain cereals are higher in phosphate than refined varieties, they need not be restricted as the phosphate is phytate-bound, and the inclusion of fiber in the diet is beneficial. The phosphate content of plant-based foods can be reduced by soaking, rehydrating, and boiling. Table 3 provides a list of foods with lower phosphate content.

If avoidance of phosphate additives does not control serum phosphate levels sufficiently, fresh foods naturally high in phosphate (milk, cheese, yogurt, eggs, meat, fish, and nuts) may need to be reduced. These high phosphate, high nutritional value foods, which provide important protein in the diet, are preferable to low nutritional value items such as cola drinks and chocolate. In more advanced CKD, phosphate binders may be required in addition to dietary phosphate restriction. Binders should be taken in conjunction with phosphate-containing meals and snacks.

For children reliant on enteral tube feeds whose phosphate intake needs to be reduced, a pediatric formula can be mixed with a whey-dominant infant formula or specialized renal-specific low potassium formula which also has a lower phosphate content. Renal-specific formulas should not be used as the sole source of nutrition due to their low potassium content which may cause a precipitous fall in serum potassium. Supplementary Table 4 provides the composition of typical renal-specific formulas. For overnight continuous EF, phosphate binders may be added to the formula before administration, but some may cause ingredients to settle out. In this case, the binder needs to be mixed with some water and put down the feeding tube (or taken by mouth) just before and/or immediately after the feeding period. Alternatively, the binder may be added to the formula and then the treated formula decanted and fed.

In the case of hypophosphatemia, dietary phosphate should be increased to achieve age-appropriate serum phosphate levels. The most effective way to do this is to offer more foods naturally high in phosphate; phosphate supplements may be necessary if this approach is not tolerated or does not correct the hypophosphatemia.

### Potassium

While potassium excretion is not usually affected until GFR is <15–20 mL/min/1.73 m^2^, hyperkalemia can develop in moderate CKD, especially as potassium intake increases with age [18]. Hyperkalemia can result from tissue breakdown in the catabolic child [22]; the latter can be resolved by giving energy supplements (Supplementary Tables 1, 3).

There are no data on the potassium requirements for children with CKD, probably due to the large variability in kidney excretion. The PRNT recommends that dietary potassium intake is only adjusted if the serum potassium level is outside the normal range, 3.5–5.0 mmol/L in children and adolescents, based on serial measurements [50]. The amount of potassium consumed can be considerably decreased by the avoidance of processed foods containing potassium additives. These are commonly added to meat and fish products, bakery items, milk puddings, ice creams, some confectionery items, and soft drinks. Educating children and their caregivers to scrutinize food labels and avoid products featuring “potassium” in the ingredient list is pivotal. As with phosphate, these inorganic food additives have high (90–100%) bioavailability [63, 64] and are an unnecessary source of dietary potassium.

In comparison, the bioavailability of potassium from animal foods and fruit juices is up to 70%, while that in plant cells in unprocessed plant foods is no greater than 50–60% [65–67]. Vegetables, pulses, and fruits have been traditionally targeted as undesirable when treating hyperkalemia due to their high potassium content. However, they should not be routinely omitted from the diet simply based on their potassium content; bioavailability needs to be considered as well. These foods offer other nutritional benefits, such as vitamins, minerals, flavonoids, polyphenols, and fiber. This is supported by the 2020 KDOQI update on nutrition in CKD [68], the Kidney Disease Improving Global Outcomes report on dyskalemia [69], and the PRNT [50]. In the past, refined grains have been favored over whole grain varieties due to their lower potassium content, but the bioavailability of potassium may be higher. Considering that whole grains contain essential nutrients and dietary fiber, they are now recommended over refined grains. It may be beneficial to choose foods with a low potassium-to-fiber ratio to enable a higher fiber intake to be maintained while lowering dietary potassium [70]. Table 3 shows lower potassium foods to offer the child if serum potassium levels are raised, despite cutting out potassium additives. Families may find it helpful to have lists of foods with low, moderate, and high potassium content, each with a daily allowance, with the caveat that these categories simply show potassium content without consideration of bioavailability. The use of a potassium “binder,” such as sodium zirconium cyclosilicate (SZC), may allow more plant-based high-fiber foods to be included in the diet and has been shown to be safe and effective in the management of acute and chronic hyperkalemia in children with advanced CKD [71]. However, as an ion exchange resin, SZC exchanges potassium for sodium so could give the child a high sodium load; there was no difference in serum sodium, blood pressure, or interdialytic weight gain seen with its long-term use.

A change in cooking methods can reduce the potassium content of foods [50]. Cooking potatoes and other tuberous roots in ample water reduces potassium content by 35–80% while soaking the raw food has very little effect. Cutting potatoes into small pieces and then double-cooking (bringing the water to a boil and then replacing it with fresh water) reduces the potassium by 50%. The potassium content of dried pulses can be reduced by up to 80% through soaking them in water for 12 h and then boiling them for 30 min and discarding the water prior to subsequent cooking. Microwave cooking reduces the potassium content of foods but to a lesser extent than boiling. Sous-vide cooking and frying increase the potassium content of foods. A dietitian should advise caregivers about cooking methods as these may reduce the amounts of other minerals and water-soluble vitamins.

When managing hyperkalemia in the child receiving enteral tube feeds, a standard pediatric formula can be mixed with a whey-dominant infant formula or specialized renal-specific low potassium formula to lower potassium content. The composition of infant and pediatric renal-specific formulas is given in Supplementary Table 4. If an adult renal-specific low-potassium formula is used, the nutritional profile needs to be carefully checked for its suitability. A low potassium formula should only be used as the sole source of nutrition in the short term (hours rather than days) as the low potassium content may cause a rapid fall in serum potassium. If used solely in the initial treatment of moderate to severe hyperkalemia, a standard pediatric formula should be introduced as soon as serum potassium levels allow.

In the case of hypokalemia, the initial approach is to address any underlying medical causes, such as excessive dialysate potassium losses, medications (potassium binding resins, diuretics), gastrointestinal losses (vomiting, diarrhea), or metabolic alkalosis. Severe, life-threatening hypokalemia requires prompt medical intervention, usually with intravenous potassium. If there is persistent hypokalemia, dietary potassium intake should be increased by including higher potassium-containing foods.

### Sodium, acid-base balance, and fluid

Children with renal dysplasia may have significant urinary losses of both salt and water and become salt and water depleted. Sodium is an important growth factor and salt deprivation can hinder growth, even when the intake of energy and other nutrients are adequate. This failure to gain weight can be reversed with sodium and water supplementation [72]. Young children on PD will very commonly also require sodium supplements. Acidosis is common in all causes of CKD when GFR falls below 25 mL/min/1.73 m^2^, and even mild acidosis is associated with poor growth due to the negative impact on bone metabolism and muscle synthesis. KDOQI recommends base supplementation, maintaining serum bicarbonate at 22 mmol/L or higher [23].

While dietary salt restriction is beneficial for many in the early stages of CKD, fluid retention as a consequence of eating too much salt is particularly problematic in those with CKD4–5D, leading to volume overload, edema, and hypertension, with the long-term risk of cardiovascular disease [73]. Sodium balance is the strongest independent predictor of interdialytic weight gain in children on HD. The CKiD study [74] has highlighted that salt intake in children with CKD2–3 often surpasses the recommendations set by KDOQI, with “fast foods” being a major contributor. Avoidance of processed foods is strongly advised with home-prepared fresh foods preferred as long as salt is not used in cooking or added at the table. Meals can be made more flavorful by adding herbs and spices. Foods to avoid when lowering the salt content of the diet are shown in Table 3. If the family uses processed foods, they should be educated to read food labels and choose those with a salt content <1.0 g salt per 100 g food. Some medications, like sodium-based potassium exchange resins and sodium bicarbonate, can be important sources of sodium.

## Transition to adult services

The ethos of holistic care must continue with the transition to adult healthcare, a particularly challenging time for young adults with CKD. The majority of patients with CKD in such units are elderly, and care is tailored towards the older patient; young adults may find that their nutritional needs are not addressed. Given that the etiology of their disease may differ to those with adult-onset kidney failure, and as bone mineralization continues into their 30s, their calcium and phosphate requirements may be considerably higher than for older adults. In addition, the young adult may have different eating habits and some may still require supplementary enteral tube feeding, particularly those with developmental delay; others may struggle with obesity and metabolic syndrome. Moreover, young adults may be living away from home without the support of caregivers. The transition process to adult services should start early and include frequent reviews by both the pediatric and adult teams, with active collaboration between dietitians from both teams. Growth, neurocognitive development, and oral development are all factors that the adult clinician should consider in the young adult, and the implications of these in their long-term nutritional care [75]. The adolescent must be strongly encouraged to take an active role in managing their nutrition before they enter the adult service.

## Key summary points

1. Ideally, children with CKD should receive care from a center with a pediatric kidney dietitian working closely with the medical team to ensure optimal nutritional management.
2. It is important to consider the many nutrition-related needs of the individual, including their nutritional requirements, their food preferences, and the eating habits of the family.
3. Prioritizing good nutrition to meet energy and protein needs is of utmost importance for achieving optimal growth in children with CKD2–5D.
4. Children should have a nutritionally balanced diet, encouraging plant-based proteins, and starchy carbohydrates, including whole grains where possible.
5. Meat, fish, eggs, milk, and dairy products can be included and are important sources of protein, vitamins, and minerals; their high phosphate content may need to be managed with phosphate binders.
6. Prompt intervention is imperative when the child cannot take sufficient food to maintain growth, with the options of food supplementation and enteral tube feeding.
7. Any dietary modification should take into account the overall nutritional adequacy of the diet.
8. The most effective strategy to reduce potassium, phosphate, and salt in the diet, while allowing greater flexibility to incorporate fresh foods, is to avoid processed foods with potassium and phosphate additives and excess salt.
9. The bioavailability of potassium from pulses, vegetables, and fruits is low; hence, these foods should not be avoided solely on account of their potassium content.
10. The bioavailability of phosphate from pulses, vegetables, and whole grains is low; hence, these foods should not be avoided solely on account of their phosphate content.
11. Salt restriction is beneficial for most children, but those with salt-losing polyuria and young children on dialysis may need a higher sodium intake to account for increased losses.
12. Transition to adult care should be made in partnership with the adult kidney center, with a focus of empowering young adults to become independent and well-informed about their dietary and medical management.

## Multiple choice questions

Choose one answer. Answers are given following the reference list.

1. To promote optimal growth, what are the energy and protein requirements for children with CKD?
  - 50% higher energy and protein than requirements for healthy children
  - Energy and protein towards the higher end of the Suggested Dietary Intake
  - Protein intake reduced to the minimal safe level while maximising energy intake
2. Many factors affect a child’s dietary intake. Which of the following statements isn’t true.
  - Acidosis, anaemia and uraemia do not influence oral intake
  - A child’s appetite for food may be impacted by gastroesophageal reflux and gut dysmotility
  - Children have reduced smell and taste sensations
3. When does a child’s dietary potassium intake need to be restricted?
  - As soon as they commence hemodialysis
  - If they have stage 3 CKD
  - When their serum potassium levels are above the normal range for age
4. If a dietary phosphate restriction is required, which foods should first be avoided?
  - Foods containing phosphate additives
  - Animal-based foods, such as milk and meat
  - High fiber foods, such as whole grains
5. When considering salt, which statement is true?
  - A salt supplement should be given to all children with CKD to promote growth
  - Eating too much salt can lead to fluid retention in children with CKD4–5D
  - Processed foods can be eaten freely when following a salt-restricted diet

## Supplementary Information

Below is the link to the electronic supplementary material.

Graphical abstract (PPTX 195 KB)

Supplementary file2 (DOCX 18 KB)

## Declarations

### Disclaimer

The Pediatric Renal Nutrition Taskforce wishes to confirm that Vitaflo has not influenced the content of this Educational Review.
