# Supplementary material for: Nutritional management of the child with chronic kidney disease and on dialysis
Source: Pediatr Nephrol. 2024 Jul 10;40(1):69–84. doi: 10.1007/s00467-024-06444-z (PMC11584487; doi:10.1007/s00467-024-06444-z)
Supplement: Supplementary file 1 — Graphical abstract (PPTX 195 KB) [file 467_2024_6444_MOESM1_ESM.pptx]

## Slide 1
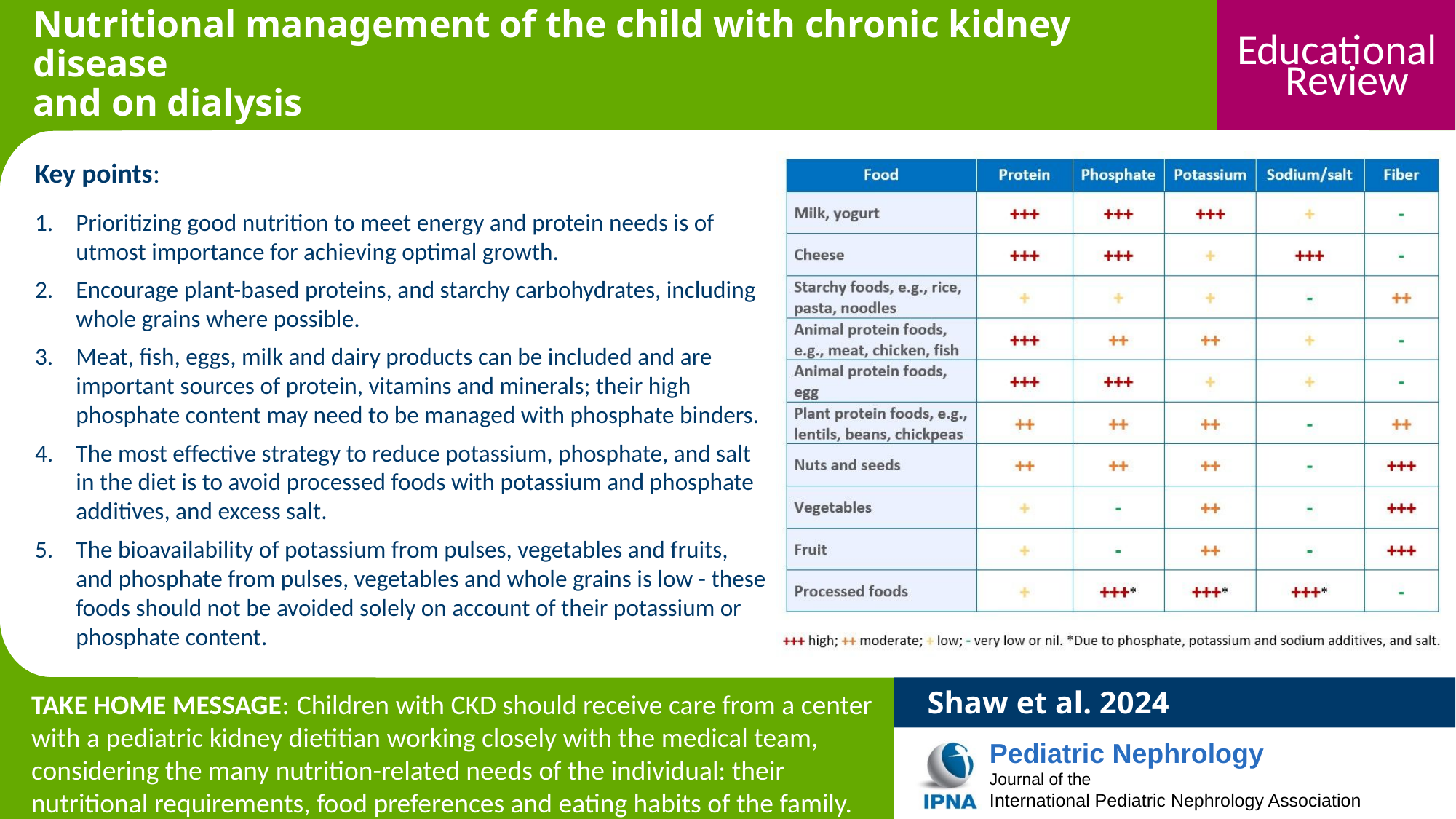

Nutritional management of the child with chronic kidney disease
and on dialysis
Key points:
Prioritizing good nutrition to meet energy and protein needs is of utmost importance for achieving optimal growth.
Encourage plant-based proteins, and starchy carbohydrates, including whole grains where possible.
Meat, fish, eggs, milk and dairy products can be included and are important sources of protein, vitamins and minerals; their high phosphate content may need to be managed with phosphate binders.
The most effective strategy to reduce potassium, phosphate, and salt in the diet is to avoid processed foods with potassium and phosphate additives, and excess salt.
The bioavailability of potassium from pulses, vegetables and fruits, and phosphate from pulses, vegetables and whole grains is low - these foods should not be avoided solely on account of their potassium or phosphate content.
Consider including a representative figure or table from your Educational Review article, if relevant, and if you have the requisite permissions.
Shaw et al. 2024
TAKE HOME MESSAGE: Children with CKD should receive care from a center with a pediatric kidney dietitian working closely with the medical team, considering the many nutrition-related needs of the individual: their nutritional requirements, food preferences and eating habits of the family.
