# Supplementary material for: Nutritional management of the child with chronic kidney disease and on dialysis
Source: Pediatr Nephrol. 2024 Jul 10;40(1):69–84. doi: 10.1007/s00467-024-06444-z (PMC11584487; doi:10.1007/s00467-024-06444-z)
Supplement: Supplementary file 2 — Supplementary file2 (DOCX 18 KB) [file 467_2024_6444_MOESM2_ESM.docx]

**Nutritional management of the child with chronic kidney disease and on dialysis**

**Supplementary tables**

**Supplementary Table 1.** Adding extra energy to foods and drinks

| **Energy supplement** | **How to use** |
| --- | --- |
| Glucose polymer powders and combined fat/glucose polymer powders – have a neutral taste and dissolve easily in ‘liquid’ or moist foods | Add to sweet foods such as porridge and other hot breakfast cereals, soft desserts such as custards and mousses  Add to savoury foods such as soup, mashed potato; vegetable, bean, and lentil dishes |
| Sugar, glucose, jams, honey or syrups – will impart a sweet taste which may limit their use | Add to breakfast cereals, desserts; spread on bread, toast, pancakes, crumpets, buns and scones |
| Fats such as vegetable spreads and oils – choose those with a high content of omega-3 fats (soya, walnut, linseed) or high in monounsaturated fat (olive oil) | Add to pulses, vegetables, rice, pasta, couscous, millet, yam and potatoes  Spread on bread, toast, pancakes, crumpets, buns and scones |
| Glucose polymer powders - can be added to beverages at high concentrations without an osmotic effect on the gut  Combined fat/glucose polymer powders - they impart a white color and a ‘milky’ mouth feel | Add to plain water, water with fruit flavorings/cordials, carbonated drinks/sodas  Start with 5% (5 g added to 100 mL) and gradually increase to 30% (30 g added to 100 mL), as tolerated |
| Sugar and glucose may be added to drinks, but the quantity may be limited due to their sweet taste and osmotic effect on the gut |  |

**Supplementary Table 2.** Enteral tube feeding formulas – typical composition per 100 mL

| **Formula** | **Energy**  **(kcal)** | **Protein**  **(g)** | **Na**  **(mg)** | **K**  **(mg)** | **Ca**  **(mg)** | **PO_4_**  **(mg)** |
| --- | --- | --- | --- | --- | --- | --- |
| Standard 13% concentration infant formula | 66 | 1.3 | 17 | 69 | 51 | 24 |
| Standard 20% concentration infant formula | 102 | 2.0 | 26 | 106 | 78 | 37 |
| Energy and nutrient dense infant formula | 100 | 2.6 | 37 | 95 | 100 | 50 |
| Standard pediatric enteral formula | 100 | 2.8 | 60 | 110 | 60 | 50 |
| High energy pediatric formula | 150 | 4.2 | 60 | 135 | 84 | 80 |
| Standard adult enteral formula | 100 | 4.0 | 88 | 148 | 68 | 68 |

**Supplementary Table 3.** Addition of energy to standard pediatric 1 kcal/mL enteral formula

| **Energy module** | **Amount of CHO/fat added to formula per 100 mL** | **Final concentration of CHO/fat in formula (g/100 mL)** | **Energy boost per 100 mL** |
| --- | --- | --- | --- |
| Glucose polymer | 8–18 g (plus 12 g CHO from pediatric formula) | 20–30 | From 100 kcal to 132–172 kcal |
| Fat emulsion (50% fat content) | 9 mL (plus 4.5 g fat from pediatric formula) | 9 | From 100 kcal to 140 kcal |

*CHO*, carbohydrate

If glucose polymer is unavailable, glucose or sucrose could be added at a suggested dose of 4 g/100 mL formula; beware increased osmolality.

If fat emulsion is unavailable, polyunsaturated or monounsaturated oil could be added at a suggested dose of 4 mL/100 mL formula; they have little effect on osmolality but the oil settles out.

**Supplementary Table 4.** Specialized renal-specific formulas – typical composition per 100 mL

|  | **Energy (kcal)** | **Protein (g)** | **Na (mg)** | **K (mg)** | **Ca (mg)** | **PO_4_ (mg)** |
| --- | --- | --- | --- | --- | --- | --- |
| 20 g Low calcium infant formula plus cooled boiled water up to 100 mL | 102 | 3.0 | 45 | 93 | <6 | 35 |
| 20 g Renal-specific low potassium infant formula plus cooled boiled water up to 100 mL | 100 | 1.5 | 48 | 22 | 24 | 19 |
| Renal-specific low potassium pediatric formula | 200 | 4.0 | 84 | 35 | 47 | 35 |
| Renal-specific low potassium adult formula | 200 | 7.3 | 68 | 24 | 5 | 6 |
| Renal-specific adult formula | 180 | 8.1 | 70 | 106 | 106 | 72 |

Supplementary Table 2 shows the composition of standard infant formula at 20% concentration for comparison.
